# The use of remote video directly observed therapy to improve both inhaler technique and adherence to asthma medications

**DOI:** 10.3389/fpubh.2022.965629

**Published:** 2022-10-05

**Authors:** Paddy McCrossan, Dara O'Donoghue, James Charles McElnay, Michael D. Shields

**Affiliations:** ^1^Centre for Medical Education, Queen's University Belfast, Belfast, United Kingdom; ^2^Royal Belfast Hospital of Sick Children, Belfast Health and Social Care Trust, Belfast, United Kingdom; ^3^School of Pharmacy, Queen's University Belfast, Belfast, United Kingdom; ^4^Centre for Experimental Medicine, Queen's University Belfast, Belfast, United Kingdom

**Keywords:** remote, asthma, video, directly, observed, therapy

## Abstract

Incorrect inhaler technique and non-adherence to inhaled preventer therapy often is the cause of poorly controlled asthma. Detecting and correcting non-adherence in asthma therapy has proven difficult. In addition, while patients may be able to demonstrate correct inhaler technique at the clinic recent evidence suggests that critical errors in inhaler technique occur in the home setting. Remote video directly observed therapy (vDOT) has recently been described as a potentially useful tool for addressing non-adherence while also allowing timely correction of inhaler technique errors. In this mini-review we describe the use of vDOT in asthma management.

## Introduction

Asthma is the most common childhood chronic illness and, in the elderly, either asthma or Chronic Obstructive Pulmonary Disease (COPD) is diagnosed in 21% of the population (1, 2). Chronic inflammation of the airways plays a central role in the pathogenesis of asthma, leading to reversible airway obstruction and airway hyper-responsiveness (2). Day-to-day wheezing, cough, and shortness of breath, along with acute episodes of wheeze triggered by respiratory viral infection or aeroallergen exposure, are typical of asthma and can significantly affect quality of life. Acute asthma attacks can be severe and life threatening, particularly if the asthma is not managed appropriately.

The mainstay of asthma management includes the avoidance, where possible, of the known triggers (which increase airways inflammation) and the regular use of an inhaled corticosteroid (ICS). ICS therapy is delivered as an aerosol to the airway surface via an inhaler and acts as a preventative medication by dampening the inflammation within the airways. ICS therapy not only helps to control milder day-to-day symptoms but also reduces the risk of future asthma attacks. To be effective, ICS must be taken correctly (correct inhaler technique) and consistently, taken once or twice daily (good adherence). It is apparent that only about one third of asthmatics use their inhaler correctly (3) and only half use their preventer inhaler regularly (4). As a result, many asthmatics have poor control and are at risk of severe asthma attacks. Thus, suboptimal inhaler use has cost implications to both the patient and the health service.

It is recognized that children with asthma and the elderly with either COPD or asthma find inhalers difficult to use (5). This problem is compounded by the wide range of inhaler devices available, each requiring slightly different inhaler technique to ensure optimal drug delivery to the airways. Patients with difficult to control asthma may require more than one inhaler type. It may therefore become even more difficult to maintain competence in using multiple devices (6).

Electronic inhaler monitoring devices (EIMDs) have become available and have been shown to be effective in improving adherence. These involve physical attachments to the inhaler which can determine whether the device has been actuated and record the date and time of each actuation. These EIMDs may be linked with Smart Phones to give patient feedback and reminders if they have forgotten to use their inhaler (7).

More recently, EIMDs that detect the acoustics of inspiratory airflow have been developed [e.g the INCA device (8)]. This addition is a step toward enabling the clinician who is monitoring the patient to know whether a satisfactory inspiratory maneuver has occurred. However, assessing inhaler technique using EIMDs remains limited as a comprehensive assessment of inhaler technique is more complex than just measuring inspiratory flow. As well as being expensive, a further significant barrier to the use of EIMDs is that they are device-specific and are not available for all types of inhaler.

There is therefore the need to develop a better solution that can improve adherence and inhaler technique simultaneously in a reliable, convenient, accessible, and cost-effective way.

## Directly observed therapy

Directly observed therapy (DOT), whereby patients are observed in taking their medication by a healthcare professional in-person, is an effective means of ensuring that they are adhering to their prescribed treatment plan. DOT has been used most extensively in the treatment of tuberculosis (TB) (9), where poor adherence increases the risk of developing multi-drug resistant strains of TB thus increasing the risk of adverse patient outcomes.

Within the context of asthma, DOT can combine an assessment of adherence with monitoring inhaler technique (with correction of technique as required). DOT has shown promising results with regards to short-term adherence in asthma (10) and has been shown to be an effective means of reducing asthma exacerbations (11).

## Video directly observed therapy

Based on the success of DOT, remote methods of delivering this approach have been developed to increase its scalability. The use of video directly observed therapy (vDOT), which involves a patient taking a video (usually taken using a mobile phone) of themselves taking their medication and submitting it for review by a healthcare professional, is now endorsed by the World Health Organization for the management of TB (12, 13).

Video directly observed therapy has also been used for the management of a broader spectrum of conditions including: opioid replacement for those suffering from addiction (14), pediatric organ transplant recipients (15), and for the use of hydroxyurea in treatment of sickle cell disease in children. In the Sickle Cell Disease study, vDOT was used to validate previously used methods of assessing adherence and found that the traditional methods were often inadequate (16).

This highlights the potential of vDOT as a tool, not only for improving treatment outcomes, but also for research purposes, to ensure that adherence is accurately monitored.

### How has vDOT been used in asthma?

Remote mobile vDOT for asthma involves the patient (or in the case of young children a carer) using their Smart Phone to capture audio and video footage of them using their inhaler, uploading the video, and forwarding it to a secure repository. The videos are time and date stamped. This allows a trained clinician to review the videos and provide personalized feedback to improve inhaler technique and encourage adherence; this approach has been shown to be effective (17). When checking a patient's inhaler technique, clinicians integrate visual observation with the sound of inhalation to determine whether correct inhaler technique has been used. Importantly, the audio component of vDOT videos allows an assessment of the inspiratory flow to be made and in the case of pressurized aerosol inhalers the release of the medication from the inhaler device.

Some vDOT platforms only allow the direct care clinical team to review the videos (www.continga.co.uk) while another platform also offers the service of a trained external observer to review the videos (www.emocha.com).

Interestingly, the use of vDOT in asthma has shown that, although many children can demonstrate correct inhaler technique at the clinic, major errors with inhaler use still occur when done at home (17). This has been under-appreciated and may result in children being wrongly diagnosed as having Severe Therapy Resistant Asthma (STRA) and treated with expensive biologic therapies when basic measures such as optimally using their preventer inhaler could have resulted in improved asthma control.

The team at the Royal Belfast Hospital for Sick Children (RBHSC) in Belfast have been using vDOT as part of the pathway for the management of difficult to treat asthma (DTA) to ensure patients have optimal inhaler technique and adherence over a period of 6–8 weeks before stepping up therapy is considered. Using vDOT helps to differentiate true STRA from apparent Difficult to Treat Asthma (DTA), which often has a remedial explanation (such as incorrect inhaler technique and/or inadequate adherence). The use of vDOT at the regional DTA clinic has significantly reduced the number of children requiring step up to expensive biologic therapies for asthma (such as anti-IgE and anti-IL5 therapies). In the 48 months before the full implementation of vDOT, some 17 asthma children within Northern Ireland were escalated to biologic therapies, while over the 48 months with vDOT implemented, that number had dropped to two patients (12).

In addition, the RBHSC team use vDOT to facilitate the FeNO suppression test (12). The FeNO suppression test is a tool which can help detect non-adherence to ICS as the cause of poor asthma control. Fractional exhaled nitric oxide (FeNO) is a non-invasive surrogate marker of eosinophilic allergic airway inflammation and is often elevated in ICS naïve patients (13). Typically, when ICS therapy is commenced, the FeNO level normalizes within 7 days. When a patient with poorly controlled asthma attends the clinic and is found to have an elevated FeNO the suppression test can be conducted. The RBHSC team use vDOT to ensure that the ICS has been taken regularly and correctly over the following 7 days. After 7 days a significant drop in the FeNO level suggests that prior non-adherence and/or incorrect inhaler technique is the likely cause for the poor asthma control.

### Ease of use for patient

In one study of vDOT for inhaler use in children, parents reported enthusiasm for the application and older children mentioned that using the app was “fun and enjoyable” (17).

A second feasibility study of vDOT for children with asthma reported that participants experienced the program as long (in their case the period of vDOT was 8 weeks), but easy to use. The benefits included building routines, improving skill, and independence (18).

In a mixed methods randomized study investigating different types of electronic monitoring devices for children with asthma, one of the four arms (*n* = 14) was vDOT. Seven patients completed focus groups. One patient reported that it was more effort than other methods (EIMD) as it sometimes required two people to take the video, and this may affect routine. VDOT was also considered by one parent to be an invasion of their privacy and had concerns around being judged on their personal appearance (7). Two of the EIMDs used in this study were deemed easier to integrate into the patient's daily routine. In addition, one patient reported using their inhaler but failing to record and submit a video. Overall, however, the feedback provided was that vDOT improved inhaler technique.

### Ease of use for clinician

One of the advantages of vDOT from the clinician's perspective is that it is inhaler device-independent (7) and allows the clinician to observe exactly what errors in inhaler technique a patient may be making. Clinicians have also recognized that vDOT is better for checking and improving inhaler technique compared with other methods of remote inhaler monitoring (7).

However, setting up and “running” the vDOT programme (without external video review and reporting) does bring an extra workload for the clinician. The patient will likely need assistance in downloading the app and setting up an account. The clinicians then must review two videos per day for each patient that they are monitoring on the app. Viewing of the videos by the clinician does not need to be performed immediately (synchronously) but rather uploaded videos can be viewed at a later convenient time (asynchronously). In RBHSC team's experience, healthcare professionals (HCPs; usually the asthma nurse) can accommodate the viewing of videos and providing timely feedback on the vDOT platform for up to four or five patients at any one time within his/her normal daily work. Therefore, we have had to limit the number of patients enrolled on vDOT to those who have the most difficult to treat asthma. As outsourcing the reviewing and reporting of video uploads could cause data protection issues in different countries it may be possible, in the future, to use artificial intelligence to do this task and offer this as part of the service.

### vDOT data security

Using vDOT requires the storage of sensitive data (videos of patients, including children). Therefore, any vDOT platform must meet all the regulatory requirements for storing such data and must have a robust method of ensuring that all data transfer is safe and secure.

The National Health Service (NHS, UK) and other health care organizations have strict codes of practice which must be adhered to.

### Efficacy of vDOT

A recent pilot study of adult asthma/COPD patients used a computer tablet device application to provide ‘real time' remote observation of inhaler use to explain technique errors to the patient. It found that, while HCPs could effectively correct the errors, this synchronous method was not sustainable as patients do not use their inhaler during the normal working hours (19). One means of overcoming this issue is using asynchronous feedback. In the Belfast pilot study of 22 children with difficult-to-treat asthma attending the RBHSC, patients uploaded twice daily videos of inhaler use (ICS) to the vDOT platform. The following day, a nurse specialist reviewed the uploaded videos and critiqued the inhaler technique. Patient reminders and the nurse feedback encouraged adherence while enrolled in the program. Of the 22 participants, 18 remained enrolled by week 5. All participants had effective and correct inhaler technique at that time. While spirometry values did not change there was a significant improvement in FeNO and ACT (Asthma Control Test) scores (17).

A second study of children with sub-optimally controlled asthma was piloted in the USA using a different vDOT platform, Emocha^®^ to that used at the RBHSC (Continga^®^). Patients uploaded videos of inhaler use and received asynchronous feedback. This pilot study suggested that use of this platform was acceptable to this population. Inhaler technique could be improved if patients continued to engage beyond 1 week but retention was low at about 50% over 8 weeks and users felt like this program was too long (18).

A further pilot study from India of newly diagnosed adult asthma/COPD patients, in a primary care setting, used WhatsApp to capture videos of patient inhaler use. Having been trained to mastery in the clinic, inhaler technique was seen to drop steadily over time (days 1, 7, 14, and 28). However, the method of remotely observing inhaler technique *via* videos provided a means to intervene to correct and prevent inhaler technique errors (20).

For any adherence tool to be effective in asthma care, it needs to be paired with strategies to change patient behavior in terms of encouraging and ensuring that they regularly take their preventer inhaler, using good technique. The engagement that vDOT encourages (between patient and clinician) provides a real opportunity for this behavioral change and provides certainty around knowledge that preventer medication has been delivered. This certainty is helpful to inform the clinician's decision on whether to step up therapy when faced with a patient whose asthma remains uncontrolled.

## What's in store for the future?

To support the previously published findings of pilot studies with vDOT, there are clinical trials using both the Emocha^®^ platform [ClinicalTrials.gov Identifier: NCT05120323] and the Continga platform in the pipeline. These studies will use a similar approach by randomizing children with asthma to either vDOT or “standard care.” Their asthma clinical outcomes, including inhaler technique, adherence, acute attacks, and lung function will be monitored.

The RBHSC team, currently reserve use of the Continga vDOT platform for those patients at most risk (“difficult-to-treat-asthma”). However, all patients with asthma (from first diagnosis to established disease) could potentially benefit from intermittent periods of vDOT monitoring since improved outcomes are generally achievable in patients who establish good adherence and appropriate inhaler technique.

Over time, adherence to ICS and inhaler technique are known to deteriorate. Sustained adherence with correct inhaler technique has proved difficult for many patients. Future research should determine the duration of vDOT needed to produce sustained benefit, bearing in mind that prolonged periods may not be well tolerated by patients. It may be that repeated short refresher periods of monitoring using vDOT may be the best approach.

Treatment of chronic disease often requires long-term use of pharmacotherapy, however, it is well described that only 50% of patients take their medications correctly and furthermore. deviations in patient adherence in clinical trials can lead to bias and significant difficulty in interpreting results. vDOT has the potential to improve the universal adherence challenge in pharmacotherapy, particularly with medicines with a narrow therapeutic index and for complex regimens (such as inhaled therapies). vDOT could also provide quantitative evidence to regulators that trial participants adhered to the therapy protocol.

## Summary

Video directly observed therapy has developed in recent years with growing evidence of improving both inhaler technique and adherence with subsequent improvement in clinical outcomes.

It has been successfully used to assess and optimize treatment for children with poorly controlled asthma. Long-term clinical outcome data are not yet available but clinical trials are currently in progress.

## Author contributions

PM produced the first draft of this mini review. DO'D, JM, and MS made suggestions and changes. All authors are involved with improving the management of asthma in children and decided to write this timely mini-review.

## Funding

Funding for open access comes from department soft funds.

## Conflict of interest

Authors MS and JM co-developed the Continga platform (www.continga.co.uk) that uses vDOT to help improve asthma management. The remaining authors declare that the research was conducted in the absence of any commercial or financial relationships that could be construed as a potential conflict of interest.

## Publisher's note

All claims expressed in this article are solely those of the authors and do not necessarily represent those of their affiliated organizations, or those of the publisher, the editors and the reviewers. Any product that may be evaluated in this article, or claim that may be made by its manufacturer, is not guaranteed or endorsed by the publisher.

## References

[1] Asthma UK. What is asthma? (2019). Available online at: https://www.asthma.org.uk/advice/understanding-asthma/what-is-asthma/

[2] Pijnenburg M Carlsen KCL. Bronchial asthma. In: Eber R Midulla F, editor. ERS Handbok of Paediatric Respiratory Medicine, Vol. 1. Lausanne: The European Respiratory Society (2003), p. 1106.

[3] Kamps AW van Ewijk B Roorda RJ Brand PL. Poor inhalation technique, even after inhalation instructions, in children with asthma. Pediatr Pulmonol. (2000) 29:39–42. 10.1002/(sici)1099-0496(200001)29:1<39::aid-ppul7>3.0.co;2-g 10613785

[4] Milgrom H Bender B Ackerson L Bowrya P Smith B Rand C. Noncompliance and treatment failure in children with asthma. J Allergy Clin Immunol. (1996) 98:1051–7. 10.1016/S0091-6749(96)80190-4 8977504

[5] British National Formulary. Respiratory system, drug delivery. Available onliine at: https://bnf.nice.org.uk/treatment-summary/respiratory-system-drug-delivery.html

[6] Kaplan A van Boven JF. Switching inhalers: a practical approach to keep on UR RADAR. Pulm Ther. (2020) 6:381–92. 10.1007/s41030-020-00133-6 33051824 PMC7672131

[7] Makhecha S Chan A Pearce C Jamalzadeh A Fleming L. Novel electronic adherence monitoring devices in children with asthma: a mixed-methods study. BMJ Open Respir Res. (2020) 7:e000589. 10.1136/bmjresp-2020-000589 33154086 PMC7646352

[8] Costello R. INCA. Available online at: http://www.incadevice.com

[9] Alipanah N Jarlsberg L Miller C Linh NN Falzon D Jaramillo E. Adherence interventions and outcomes of tuberculosis treatment: a systematic review and meta-analysis of trials and observational studies. PLoS Med. (2018) 15:e1002595. 10.1371/journal.pmed.1002595 29969463 PMC6029765

[10] Halterman JS Riekert KA Fagnano M Tremblay PJ Blaakman SW Tajon R. Effect of the School-Based Asthma Care for Teens (SB-ACT) program on asthma morbidity: a 3-arm randomized controlled trial. J Asthma. (2022) 59:494–506. 10.1080/02770903.2020.1856869 33307900 PMC8285039

[11] Pertzborn MC Prabhakaran S Hardy A Baker D Robinson MA Hendeles L. Direct observed therapy of inhaled corticosteroids for asthma at school or daycare. Pediatr Allergy Immunol Pulmonol. (2018) 31:226–9. 10.1089/ped.2018.0912 30595951 PMC6306660

[12] Shields MD McElnay J. Mobile video directly observed therapy can be used to improve at-home inhaler technique in children with asthma. ERJ Open Res. (2021) 7:00463–2021. 10.1183/23120541.00463-2021 34616838 PMC8488349

[13] Coumou H Bel EH. Improving the diagnosis of eosinophilic asthma. Expert Rev Respir Med. (2016) 10:1093–103. 10.1080/17476348.2017.1236688 27624868

[14] Tsui JI Leroux BG Radick AC Schramm ZA Blalock K Labelle C. Video directly observed therapy for patients receiving office-based buprenorphine–a pilot randomized controlled trial. Drug Alcohol Depend. (2021) 227:108917. 10.1016/j.drugalcdep.2021.108917 34399136 PMC8464515

[15] Killian MO Clifford SA Gupta D. Directly observed therapy to promote medication adherence in paediatric heart transplant recipients. Cardiol Young. (2021) 31:2048–50. 10.1017/S1047951121002109 34092272

[16] Creary S Chisolm D Stanek J Neville K Garg U Hankins JS. Measuring hydroxyurea adherence by pharmacy and laboratory data compared with video observation in children with sickle cell disease. Pediatr Blood Cancer. (2020) 67:e28250. 10.1002/pbc.28250 32386106

[17] Shields MD ALQahtani F Rivey MP McElnay JC. Mobile direct observation of therapy (MDOT)-A rapid systematic review and pilot study in children with asthma. PLoS ONE. (2018) 13:e0190031. 10.1371/journal.pone.0190031 29401500 PMC5798760

[18] McIntire K Weis B Litwin Ye L Krugman S. Feasibility of video observed therapy to support controller inhaler use among children in West Baltimore. J Asthma. (2021). 10.1080/02770903.2021.1984525. [Epub ahead of print]. 34550849

[19] Trosini-Désert V Lafoeste H Regard L Malrin R Galarza-Jimenez M-A Amarilla CE. A telemedicine intervention to ensure the correct usage of inhaler devices. Telemed J E Health. (2020) 26:1336–44. 10.1089/tmj.2019.0246 32302518

[20] Dhadge N Shevade M Kale N Narke G Pathak D Barne M. Monitoring of inhaler use at home with a smartphone video application in a pilot study. NPJ Prim Care Respir Med. (2020) 30:1–6. 10.1038/s41533-020-00203-x 33067469 PMC7567806

